# Relaxation Response and Resiliency Training and Its Effect on Healthcare Resource Utilization

**DOI:** 10.1371/journal.pone.0140212

**Published:** 2015-10-13

**Authors:** James E. Stahl, Michelle L. Dossett, A. Scott LaJoie, John W. Denninger, Darshan H. Mehta, Roberta Goldman, Gregory L. Fricchione, Herbert Benson

**Affiliations:** 1 MGH Benson-Henry Institute, Massachusetts General Hospital, Boston, Massachusetts, United States of America; 2 Dartmouth-Hitchcock Medical Center, Section of General Internal Medicine, Lebanon, New Hampshire, United States of America; 3 MGH Institute for Technology Assessment, Massachusetts General Hospital, Boston, Massachusetts, United States of America; 4 MGH Division of General Medicine, Massachusetts General Hospital, Boston, Massachusetts, United States of America; 5 MGH Department of Psychiatry, Massachusetts General Hospital, Boston, Massachusetts, United States of America; 6 MGH Department of Psychiatry, University of Louisville, Health Promotion and Behavioral Sciences, Louisville, Kentucky, United States of America; Örebro University, SWEDEN

## Abstract

**Background:**

Poor psychological and physical resilience in response to stress drives a great deal of health care utilization. Mind-body interventions can reduce stress and build resiliency. The rationale for this study is therefore to estimate the effect of mind-body interventions on healthcare utilization.

**Objective:**

Estimate the effect of mind body training, specifically, the Relaxation Response Resiliency Program (3RP) on healthcare utilization.

**Design:**

Retrospective controlled cohort observational study. Setting: Major US Academic Health Network. Sample: All patients receiving 3RP at the MGH Benson-Henry Institute from 1/12/2006 to 7/1/2014 (n = 4452), controls (n = 13149) followed for a median of 4.2 years (.85–8.4 yrs). Measurements: Utilization as measured by billable encounters/year (be/yr) stratified by encounter type: clinical, imaging, laboratory and procedural, by class of chief complaint: e.g., Cardiovascular, and by site of care delivery, e.g., Emergency Department. Subgroup analysis by propensity score matched pre-intervention utilization rate.

**Results:**

At one year, total utilization for the intervention group decreased by 43% [53.5 to 30.5 be/yr] (p <0.0001). Clinical encounters decreased by 41.9% [40 to 23.2 be/yr], imaging by 50.3% [11.5 to 5.7 be/yr], lab encounters by 43.5% [9.8 to 5.6], and procedures by 21.4% [2.2 to 1.7 be/yr], all p < 0.01. The intervention group’s Emergency department (ED) visits decreased from 3.6 to 1.7/year (p<0.0001) and Hospital and Urgent care visits converged with the controls. Subgroup analysis (identically matched initial utilization rates—Intervention group: high utilizing controls) showed the intervention group significantly reduced utilization relative to the control group by: 18.3% across all functional categories, 24.7% across all site categories and 25.3% across all clinical categories.

**Conclusion:**

Mind body interventions such as 3RP have the potential to substantially reduce healthcare utilization at relatively low cost and thus can serve as key components in any population health and health care delivery system.

## Introduction

Poor psychological and physical resilience in response to stress drives a great deal of health care utilization. Stress is a mind-body phenomenon, affecting both mental health and directly influencing physiology, the course of illness and the effectiveness of disease management [1–8]. Managing stress thus is central to achieving and maintaining wellness and the appropriate use of clinical resources.

In primary care, stress-related illnesses are known drivers of healthcare resource utilization in the US [9–11]. Health care expenditures attributable to stress-related disorders, such as, depression and anxiety, were over 80 billion dollars/year in 2012. These have been the third highest cause of healthcare expenditures after heart disease and cancer (meps.ahrq.gov) in the US; each of which carries their own substantial stress burden. Over 90% of people suffering from stress or stress-related problems seek help through primary care and tend to be frequent healthcare utilizers[12]. These visits can comprise as much as 70 percent of physicians' caseloads [13]. In addition, more than 80% of patients presenting to general practice evidence lack of resiliency and psychological stress[14]. Common physical manifestations of stress, e.g., headaches, back pain, insomnia, gastroesophageal reflux disease, irritable bowel, chest discomfort, are among the most frequent reasons people seek care.

Mind body medicine focuses on the relationships between the mind and body, and on the effects changes in physiology and behavior have on health and disease. Mind body interventions are widely considered safe and can improve symptom management in a wide variety of illnesses [15–17]. Mind body modalities are frequently incorporated into treatment plans because of their low risk, mental and physical health benefits, relatively low cost, and ability to engage patients and help them take control of their health and participate as active members of the therapeutic team.

Mind body medicine programs such as the Benson-Henry Institute’s (BHI) Relaxation Response Resiliency Program (3RP)[18] and others, such as Mindfulness-Based Stress Reduction (MBSR) [19], are effective in reducing and managing the clinical manifestations of stress, reducing anxiety, and increasing patients’ resiliency[20]. The BHI 3RP specifically focuses on these areas and has demonstrated its effectiveness in reducing the need for chronic pain therapy[21], improving cardiovascular parameters[22–24], improving of anxiety and chronic stress[25,26], menopausal symptoms[27,28] and promoting positive health behaviors[29].

Previous studies in the US have suggested that mind body interventions might be cost-saving [30–32]. This may in part be due to reducing excess use of expensive and potentially unnecessary tests and procedures; actions often taken in response to risk aversion on the part of the clinician, patient anxiety and uncertainty in both[33]. In the current push toward accountable care organizations, it is prudent to reevaluate the role of mind body interventions, since they are inexpensive and have been shown improve stress-related symptoms and physiology [29,34–37], as noted, large drivers of healthcare costs. To determine whether mind body interventions can reduce healthcare utilization across a broad population of patients with a range of different health conditions, we examined the healthcare utilization of a large and diverse cohort of patients who participated in the Relaxation Response Resiliency Program offered at the Benson-Henry Institute at Massachusetts General Hospital (MGH).

## Methods

**Fig 1:**
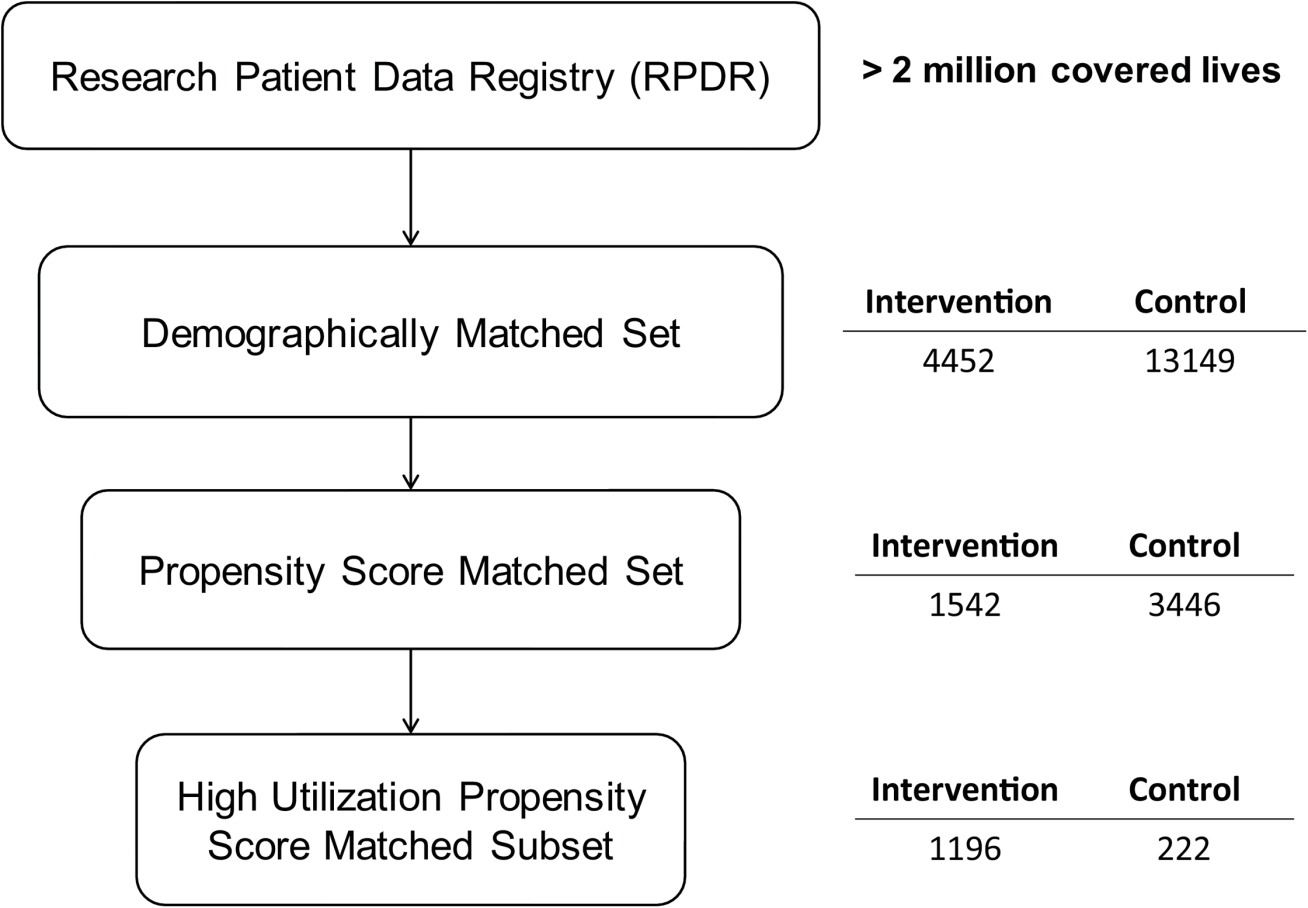
Sample selection.

A retrospective controlled cohort pre/post intervention database analysis was conducted exploring the resource utilization of all patients who presented for care at the MGH Benson-Henry Institute from 1/12/2006 to 7/1/2014 compared to controls. Patient healthcare resource utilization, 1-year before and after the date of their 3RP intervention, was compared. The control group was comprised of patients matched by age, ethnicity, and gender to the intervention group. The control group was initially set at three times the size of the intervention group. Once collected, the whole group was subjected to propensity score matching and subgroups were identified and matched by initial utilization rate. Fig 1.

The database used for this project was the Research Patient Data Registry (RPDR). The RPDR is a centralized clinical data registry, or data warehouse. The RPDR gathers data from various hospital legacy systems, which include administrative, billing, laboratory databases in the Partners network, which is centered on the Massachusetts General Hospital and the Brigham and Women’s Hospital and their affiliates, and stores it in one place. Researchers access this data using the RPDR online Query Tool with user-defined queries of RPDR data for aggregate patient totals and, with proper IRB approval, obtain detailed clinical data. The RPDR brings clinical information to the researcher and ensures the security of patient information by controlling and auditing the distribution of patient data within the guidelines of the IRB and with the use of several built-in, automated security measures. All patient records and information were anonymized and de-identified prior to analysis.

The intervention group was any patient referred to the MGH Benson Henry Institute who underwent the relaxation response and resiliency trainingin a group or individually during the study period. The 3RP intervention [18] is an integrated program of relaxation response (RR) eliciting meditation and mindfulness exercises, social support, cognitive skills training, and positive psychology[38] focused on developing skills to reduce the stress response, elicit the relaxation response and enhance resiliency[39]. The relaxation response is a hypothalamic-mediated reaction resulting in decreased sympathetic nervous system activity, decreased heart rate, lower metabolism, and decreased respiratory rate[40]. It can be considered the physiologic and psychologic opposite of the "fight or flight," or stress response and can be induced with techniques such as meditation, yoga and biofeedback among others. Resiliency is the ability to adapt well, recover quickly or thrive in response to stress, adversity, or trauma. Resiliency is cultivated in the 3RP through mindfulness training, positive psychology and cognitive behavioral training. Patients referred to the BHI often have chronic stress-related complaints which can be physical or psychological. The 3RP is conducted either in group or individual skills based settings [18] over 8 sessions.

The unit analysis for resource utilization was the billable encounter and the services associated with the billable encounter. A billable encounter is defined as a face-to-face contact between a patient and a health professional whose services are covered under an insurance provider. To count, the encounter must be recorded in the patient's health record. Services such as lab tests are billed on an encounter basis. Encounters are time stamped which allows encounters over time to be evaluated.

Because this was a retrospective study in which patients entered the healthcare system and had their interventions at different times, the intervention itself served as time 0. A new field was created in the database where all dates were recalculated relative to this time 0. Pre-intervention time was the entire time the patient was in the healthcare system before the intervention and post-intervention time was the entire time the patient was in the healthcare system commencing after the end of the intervention. This allowed us to normalize the patients’ utilization history on a common time line and aggregate it for analysis.

For the control group, time 0 was the midpoint time the individual was in the healthcare system. Pre and post intervention times were treated in the same way as the intervention group. Utilization rate was calculated in the same manner as the intervention group.

The control group and intervention group records were combined and propensity scores were generated for participation in the intervention versus control group[41]. A relatively expansive use of interaction terms for all demographic variables was used[42]. Intervention and control group members were matched to the first 5 digits of their propensity scores[43], the result was a study set with a 1:2 match of intervention:control group.

To examine the question, “Were the BHI referred patients simply high utilizers whose utilization simply regressed to the mean over time?”, we conducted further subgroup analysis in which the top tier of the control group utilizers were matched with a subset of the intervention group, where their utilization rate was greater or equal to the median utilization rate of the intervention group. The intervention group subset contained all members of the intervention group minus members whose initial maximum utilization rate exceeded the maximum utilization of the high utilizer control group members, i.e., the aforementioned top utilizers; creating two groups with the same median initial utilization rate, same maximum utilization rate and variance.

For the purposes of this analysis, the utilization rate was defined as the number of billable encounters and services used over the course of a year. Utilization rates were compared before and after within the intervention arm and before and after against the control arm. Both arms were further stratified by the type of encounter: clinical, imaging, laboratory and procedural as defined by the encounter administrative database. Both intervention and control arms were also stratified by class of the chief complaint: Cardiovascular, Dermatologic, Endocrine, ENT, Gastrointestinal, Genitourinary, Hematology/Oncology, Laboratory, Musculoskeletal, Neurologic, Obstetrics-Gynecology, Ophthalmology, Pediatric, Psychiatric, Pulmonary, Renal, and General Symptoms, such as fatigue.

Standard statistical methods were used including t-tests, ANOVA and regression methods where appropriate. JMP 11 (SAS™ product) was used for the statistical analysis.

The project was approved by the Partners Healthcare Institutional Review Board, the body providing ethics oversight for research. All patient records and information were anonymized and de-identified prior to analysis and stored on encrypted servers only accessible to the primary investigator or his institutionally approved designate.

## Results

After matching participants by age, gender and ethnicity we found that intervention patients were more likely to speak English, identify as Asian versus African-American and more likely to identify as Jewish or not having a religious affiliation. Members of the control group were slightly more likely to identify as Christian or Muslim (Table 1). Individuals in the intervention group were also less likely to be a veteran, though they were more likely to be US born. Finally, the intervention group had an approximately 10% higher median income ($77K vs. $70K). In the propensity score, high utilization matched subgroup, the intervention group was slightly more female than the group as a whole and the control group was slightly older, more Christian and more likely to be from the US and be a veteran than the group as a whole. Both intervention and control groups spent the same amount of time, greater than 4 years continuously, within the healthcare system (Table 2).

**Table 1:** Socio-demographic data.

		Population			Propensity score matched			High Utilization Subgroup analysis		
		Intervention (INT)	Control (CTL)	p diff	INT	CTL	P Diff	INT	CTL	p diff
	**N**	4452	13149		1542	3446		1196	222	
**Gender (%)**	Female	70.5	70.5	Ns	75.8	74.3	Ns	76	68	0.01
	Male	29.5	29.5	Ns	24.2	25.7	Ns	24	32	
**Age (Years)**	Mean	49.8	49.8	Ns	50.1	48.8	.004	49.7	52.7	0.005
	25%/ Med/ 75%	38/ 50/ 61	38/ 50/ 61		39/50/61	39/49/58		38/50/60	43/53/63	
**Language (%)**	English	96.7	93.9	< .05	98.2	98.2	< .05	98	99.6	Ns
	Spanish	0.5	1.7		.3	.9		1.75	.4	
	Other	2.9	4.4		1.6	.9		.25	0	
**Race (%)**	African-American	3.46	3.05	Ns	1.4	1	< .05	1.4	1.4	Ns
	Asian	3.38	3.57		1.1	.6		1.2	0	
	Caucasian	84.8	84.71		94.9	92.4		94.9	95.5	
	Hispanic	2.3	2.24		.6	1		.6	.9	
	Nat. Am./ Pac.Isl.	0.18	0.09		.1	.3		0	0	
	Other	5.89	6.33		2	4.6		1.9	2.3	
**Marital status (%)**	Married	52.8	52.6	Ns	55.1	55.3	Ns	55.2	57.7	Ns
	Not Married	47.2	47.2		44.9	44.7		44.8	42.3	
**Religion (%)**	Atheist	0.2	0.1	< .05	.19	.06	< .05	.3	.5	<0.0001
	Buddhist	0.6	0.5		0	.17		0	0	
	Christian	44.4	57.2		62.3	61.2		63.6	80.6	
	Hindu	0.6	0.4		.4	.09		.4	0	
	Islam	0.6	1.1		.32	.29		.3	0	
	Jewish	10.5	5.5		6.7	3.2		6.8	3.6	
	Other	42.9	35.2		30.1	35		28.7	15.3	
**Veteran (%)**	Yes	2.8	3.4	< .05	1.6	1.1	< .05	1.5	3.2	0.02
	No	84.8	71.7		84.7	76		88.1	91.4	
	Other	12.4	24.9		13.7	22.9		10.3	5.4	
**Country (%)**	US	69	63.2	< .05	71.1	71.1	Ns	75.2	83.3	.01
	Other*	31	36.8		28.9	28.9		24.8	16.7	
**Income ($K)**	Mean	82	76	< .05	83	78	< .05	84	77	<0.001
	25%/ Med/ 75%	64/ 77/ 99	57/ 70/ 91		65/77/99	61/74/92		65/79/99	59/72/91	

* proportion of patients with no country of origin recorded in the administrative database. of note all patients had US postal codes

**Table 2:** Patient Continuous Time in Healthcare Network.

		Mean	25% quartile	Median	75% quartile	P
Days	Intervention	1649	271	1582	3369	Ns
	Control	1613	315	1538	2943	
Years	Intervention	4.5	0.7	4.3	9.2	Ns
	Control	4.4	0.9	4.2	8.1	

The most significant result with regard to utilization was for the whole group an average 43% reduction in billable encounters for intervention patients across all functional categories (p = <0.0001) with a relative reduction of 45.1%(Table 3). For the propensity score and initial high utilization rate matched sub group this was an absolute 42.1% and 22.2% relative reduction in billable encounters. The intervention group had significant absolute reductions in all clinical encounter types (Table 4) for both the group as a whole and in the subset analysis. The control group on the other hand had general but not statistically significant increases in use of outpatient visits, specialty visits, and hospital admissions. This increase in clinical encounters was most pronounced in the specialty care settings in the control group.

**Table 3:** Functional Category: Utilization Rate (Pre- vs. Post—Intervention) by Average Billable Encounters/year.

		Whole group			High Utilization subgroup				
Category	Arm	Pre	Post	P	Pre	Post	p	% Δ Arm	% Δ I/C Ratio
Total	Intervention	53.46	30.47	*<0.0001	67.26	38.91	< .0001	-42.1	
	Control	14.76	15.32	0.1	67.58	50.28	< .0001	-25.6	
		**<0.0001	<0.0001		0.95	0.0067			***-22.2
Clinical	Intervention	39.96	23.21	<0.0001	47.65	29.26	< .0001	-38.6	
	Control	10.73	11.47	0.004	47.48	37.1	< .0001	-21.9	
		<0.0001	<0.0001		0.96	0.02			-21.4
Imaging	Intervention	11.52	5.72	<0.0001	13.46	8.05	< .0001	-40.2	
	Control	3.89	3.69	0.11	13.69	8.79	< .0001	-35.8	
		<0.0001	<0.0001		0.84	0.48			-6.9
Laboratory	Intervention	9.82	5.55	<0.0001	12.33	7.44	< .0001	-39.7	
	Control	2.62	2.52	0.29	7.8	7.53	0.79	-3.5	
		<0.0001	<0.0001		< .0001	0.92			-37.5
Procedures	Intervention	2.15	1.69	0.006	2.67	3.79	0.01	41.9	
	Control	1.66	1.52	0.43	2.52	3.71	0.27	47.5	
		0.007	0.29		0.8	0.93			-3.6

*p row is within arms across phases

**p column is within phase across arms

*** change in ratio of Intervention/Control utilization

**Table 4:** Service Site: Utilization Rate (Pre- vs. Post—Intervention) by Average Billable Encounters/year.

		Whole group			High Utilization subgroup				
Category	Arm	Pre	Post	P	Pre	Post	p	% Δ Arm	% Δ I/C ratio
General Medicine	Intervention	2.3	1.18	*0.008	4.29	3.84	0.25	-10.5	
	Control	1.79	2.24	0.21	3.38	3.77	0.59	11.5	
		**0.11	0.02		0.07	0.92			***-19.7
Specialty Care	Intervention	19.77	10.13	<0.0001	23.69	11.87	< .0001	-49.9	
	Control	3.63	3.96	0.002	8.38	8.42	0.97	0.5	
		<0.0001	<0.0001		< .0001	0.02			-50.1
Urgent Care	Intervention	2.05	0.99	<0.0001	47.24	28.96	< .0001	-38.7	
	Control	0.79	0.81	0.76	54.55	40.8	< .0001	-25.2	
		<0.0001	0.0069		0.04	0.0005			-18.0
Emergency Department	Intervention	3.61	1.67	<0.0001	2.6	1.92	< .0001	-26.2	
	Control	1.65	1.41	0.004	1.15	1.36	0.46	18.3	
		<0.0001	0.017		0.03	0.24			-37.6
Hospital Admissions	Intervention	2.3	1.18	0.008	4.29	3.84	0.25	-10.5	
	Control	1.79	2.24	0.21	3.38	3.77	0.59	11.7	
		0.11	0.02		0.07	0.92			1.8

*p row is within arms across phases

**p column is within phase across arms

*** change in ratio of Intervention/Control utilization

With regard to clinical categories (Table 5), All within pre and post phase comparisons in the comparisons between intervention and control were statistically significantly different with p<0.05. The top five presenting areas in terms of total encounters in the intervention group were: Neurologic (28%), General Symptoms (20%), Cardiovascular (11%), Gastrointestinal (11%) and Psychiatric (9%) with reductions in utilization relative to controls, i.e., in billable encounters, averaging greater than 70%, with absolute reductions within the intervention group greater than 65%. The greatest reductions in utilization were found in the Hematology/Oncology category while the least were found in Endocrine though each had less than 1% of total encounters. In the high utilization subgroup comparison, the top four presenting areas were: Neurologic (14%), Cardiovascular (11%), Musculoskeletal (10%), and Gastrointestinal (10%) with utilization reductions relative to controls of 20%, 22%, 13%, and 23% respectively. The greatest reductions were in the Hematology/oncology category the least in the genitourinary category.

**Table 5:** Disease Category: High Control Utilization subgroup vs Intervention Group.

		Pre	Post	P	Absolute Intervention Change (%)	Relative Intervention Change (%)
Cardiovascular	Intervention	100.05	35.06	< .0001		
	Control	67.76	45.06	0.016		
		0.23	0.4		-65.0	-69.8
Dermatology	Intervention	99.67	22.05	0.005		
	Control	78.33	61.33	0.66		
		0.76	0.04		-77.9	-91.3
Endocrinology	Intervention	54.54	14.38	0.004		
	Control	55.67	50.17	0.73		
		0.95	0.004		-73.6	-69.3
Gastroenterology	Intervention	78.23	32.73	< .0001		
	Control	73.85	51.15	0.24		
		0.86	0.28		-58.2	-41.9
Hematology /Oncology	Intervention	146.5	20.83	0.05		
	Control	71.11	48.51	< .0001		
		< .0001	0.06		-85.8	-163.1
Infectious Disease	Intervention	74.69	25.38	0.02		
	Control	62	27.67	0.008		
		0.004	0.02		-66.0	-28.7
Musculoskeletal	Intervention	107.15	48.7	0.02		
	Control	69.38	42.92	0.04		
		0.24	0.58		-54.5	-41.0
Neurologic	Intervention	77.77	39.48	< .0001		
	Control	62.88	49.44	0.47		
		0.51	0.68		-49.2	-43.8
Obstetrics Gynecology	Intervention	77.59	35.97	0.04		
	Control	55.72	34.94	0.001		
		0.37	0.92		-53.6	-36.3
Psychiatry	Intervention	62.84	31.21	0.001		
	Control	63.64	59.36	0.57		
		0.96	0.01		-50.3	-46.2
Pulmonary	Intervention	115.56	15.89	0.005		
	Control	71.5	77.5	0.81		
		0.37	0.001		-86.2	-141.1

When major presenting disease categories were compared, intervention patients were statistically more likely (p<0.05) to carry primary diagnoses of psychiatric, neurologic, rheumatologic and gastrointestinal conditions at the time of their intervention.

### Subgroup analysis

**Fig 2:**
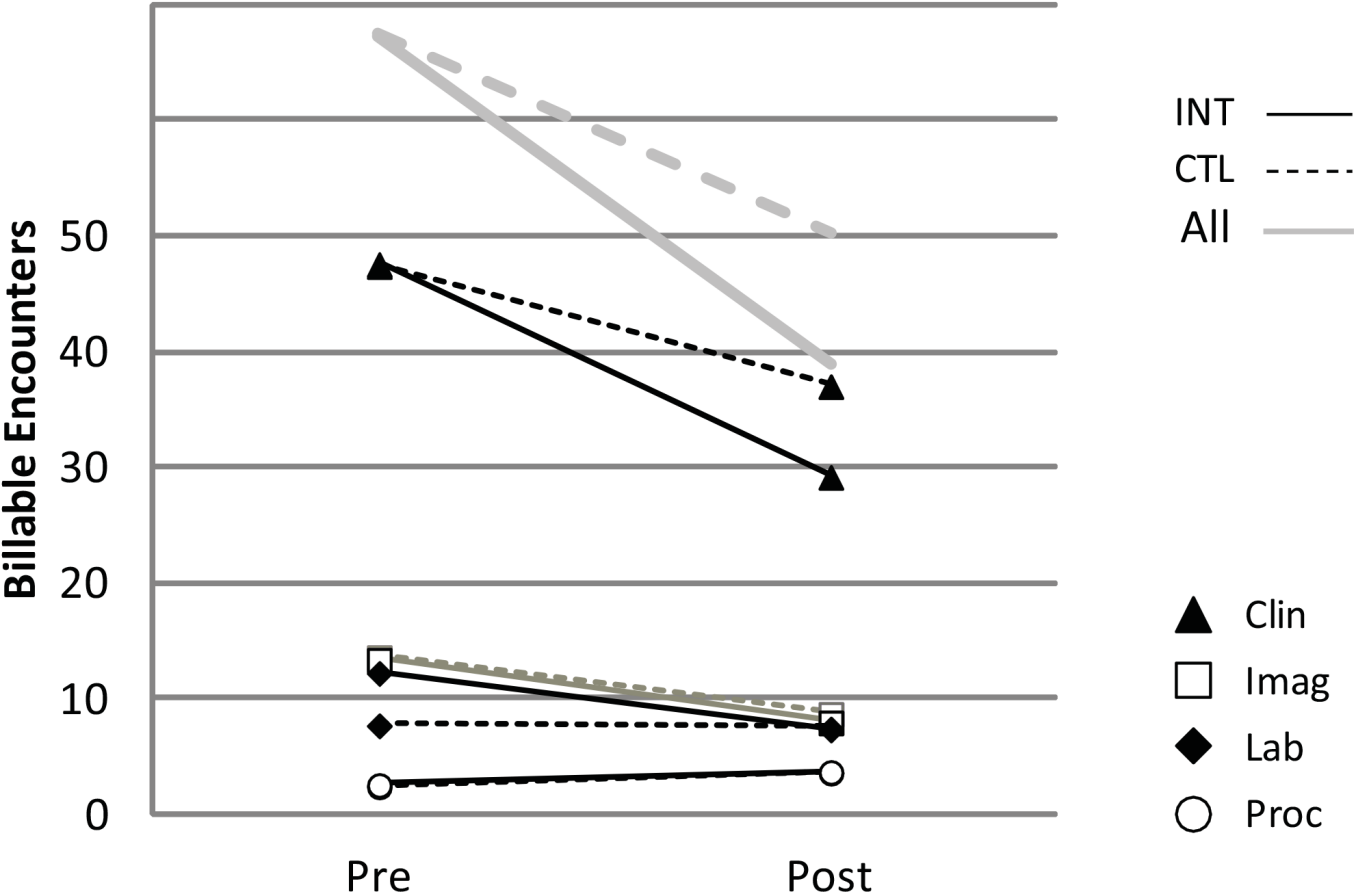
Utilization by Functional class: high utilizing controls vs. propensity score, initial utilization rate matched intervention group. INT = Intervention, CTL = Control, Clin = Clinical, Imag = Imaging, Lab = Laboratory, Proc = Procedure.

**Fig 3:**
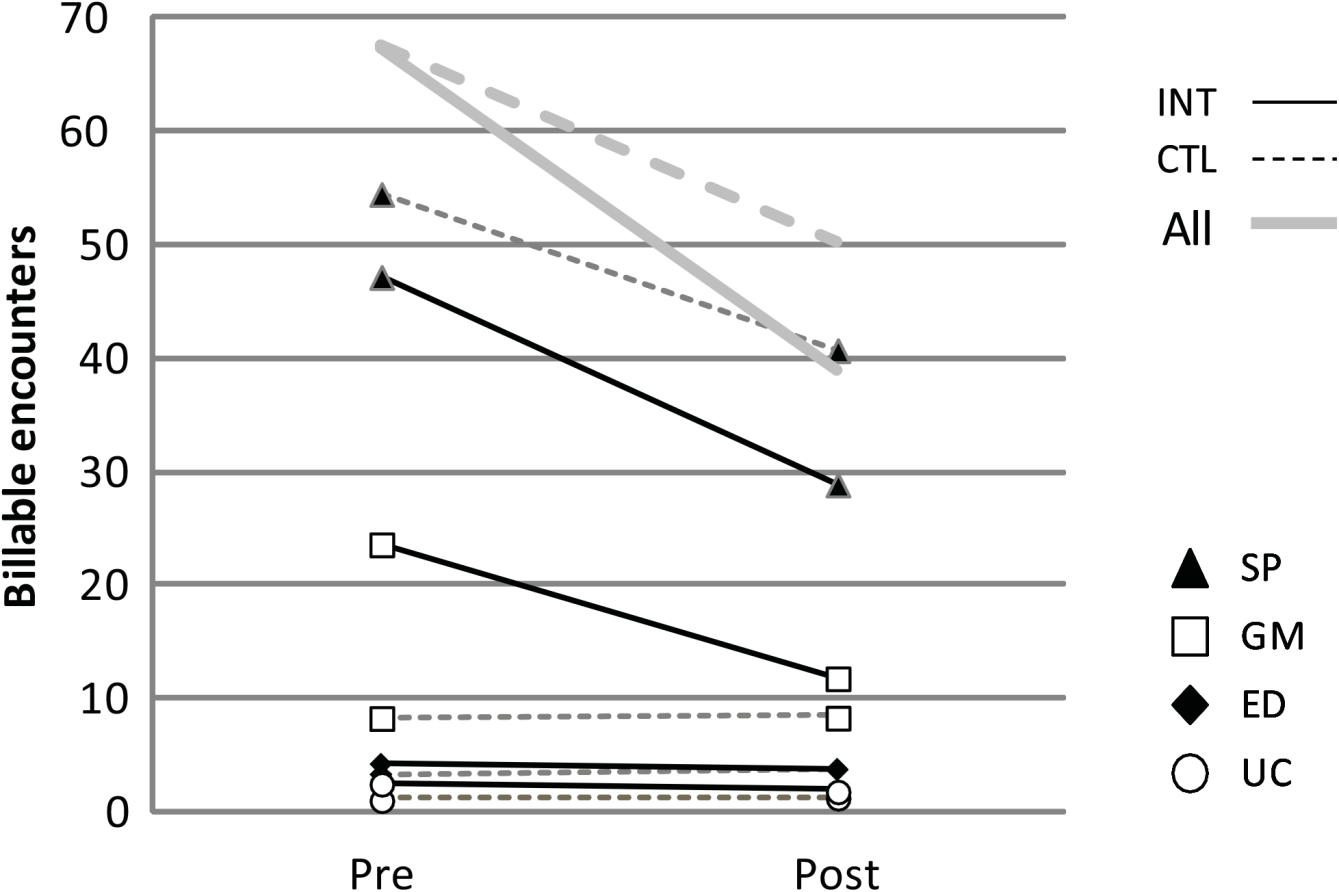
Utilization by Clinical Site: high utilizing controls vs. propensity score, initial utilization rate matched intervention group. INT = Intervention, CTL = Control, Clin = Clinical, Imag = Imaging, Lab = Laboratory, Proc = Procedure.

In subgroup analysis, in the propensity scored matched subgroup, high utilizers in the control group, matched to by utilization rate to the intervention group, did show some regression to the mean over time (Figs 2 and 3). However, the matched intervention group had statistically greater reductions in healthcare resource utilization than the control group in the post intervention period across all functional categories and all site categories except for Emergency Care (Table 4). The intervention group statistically significantly outperformed the controls with an average relative utilization reduction of: 18.3% in functional categories, a 24.7% reduction across clinical site categories.

With regard to site utilization, the intervention group had significant reductions in all categories except Hospital care. In the Urgent care category the intervention group had fewer absolute visits post intervention than the control group. In the General Medicine, Emergency department and Hospital settings, the intervention group went from significantly more use than the controls to becoming indistinguishable from the control in the post intervention period. In the Specialty care category, the intervention group had a 49.9% reduction but the final post intervention rate still remained slightly above the control group, going from 2.8 times to 1.4 times the control rate.

With regard to functional categories, the intervention group showed significant reduction in all categories, except for procedures which increased in both groups. Imaging decreased in both groups over the study period. Laboratory testing and Imaging converged becoming indistinguishable from controls in the post intervention period. Clinical encounters were 21.4% lower in the intervention group compared to controls post intervention.

With regard to disease categories the intervention group outperformed the high utilizer control group in resource use reduction in the cardiovascular, gastrointestinal, hematology/oncology, musculoskeletal, neurologic, psychiatric and pulmonary categories (Table 5).

## Discussion

The main finding of this study is that the Relaxation Response Resiliency Program (3RP) may significantly reduce healthcare utilization. This reduction is on the order of that found by Caudill[21], Group Health[13], and Kaiser Permanente[44] and other similar time-limited interventions.

The focus in healthcare is shifting from high utilization of specialized care for the treatment of late-stage disease to an emphasis on patient-centered approaches and coordinated care teams that promote wellness, support self-care, provide preventive care and effective disease management. In the process of moving from transaction-based health care to wellness and prevention based care, it is prudent to identify strategies and therapies that are both clinically effective and cost beneficial. Our results indicate that mind body interventions, such as 3RP, can reduce individual disease burden as well as the utilization of healthcare resources and are well suited to the changing healthcare environment.

Mind body medicine interventions are inexpensive relative to the cost of an emergency room visit, a hospitalization or even other complementary and alternative medicine (CAM) therapies[45]. Assuming societal values (meps.ahrq.gov) for the cost of care, the cost savings from reduced emergency room visits alone in the treatment group relative to the control group, is on the order of $2360/patient/year. This translates as a return on investment (ROI) of 4–6 months for the reduction of emergency department visits alone, based on commonly charged fees for 3RP and similar mind body programs. Of course, patients use more than emergency rooms for their care. Using an estimate, based on improvements in average utilization rates including outpatient care (general and specialist), emergency department and urgent care visits and hospitalizations, instituting 3RP could save the healthcare system substantially. Assuming median values for visits at these treatment sites gives an expected range of cost savings of $640- $25,500/patient/year; assuming average site costs gives and expected cost savings of $1500- $60,200/patient/year. The low end of these ranges assumes only the lowest cost treatment site category is used in a year and the high end assumes individual encounters in all treatment site categories in a single year. These estimates are rough and based on aggregate numbers but give a sense of the scale of the opportunities available. In the move from transactional healthcare to a more systems-based healthcare environment, these interventions may be able to significantly improve financial margins. Recent studies point toward their cost-effectiveness [30–32,46]. A more detailed large prospective cost accounting analysis is beyond the scope of this current paper but is certainly called for and is being planned.

It should be noted the public may be ahead of medical and insurance organizations in adopting these kinds of patient-centered approaches. Already, more than 10% of adults in the US report using mind body medicine tools[15]. Data from the 2012 National Health Interview Survey demonstrates that 8% of Americans had used meditation, 11% used deep breathing tools and 10% had used yoga, tai chi, or qi gong within the last year[47].

It is currently unclear how best to identify in advance patients who would be most effectively served by these interventions. Nonetheless, our analysis suggests that likely categories to explore are patients with mental health, neurologic, musculoskeletal, and gastroenterological concerns, particularly those who endorse high levels of stress. In this era of electronic medical records it may also be possible to identify other high utilizing patients as potential targeted beneficiaries.

### Limitations

As with all retrospective studies, selection bias can occur, depending on how subjects were defined and the limitations of the categories available in the database. We tried to limit this bias by oversampling our control group and matching subjects based on age, gender, and ethnicity, then using propensity score matching methods. Using any set of administrative data, however, incurs the risk of data categories that are not specifically designed for clinical studies. As a result the data may be subject to the interpretation of the person entering the data and this data may have been entered for administrative or billing purposes rather than clinical needs. However, because our focus is on utilization this shortcoming should not affect our main results.

Another limitation of this project to date is that by focusing on utilization rather than cost accounting or long term outcomes such as mortality and morbidity, which were beyond the scope of data readily available through the database used, we can only look at relative changes in use rather than performing a full cost-effectiveness analysis. The RPDR database used does not readily permit these types of calculations but based on these initial results we will be seeking resources to perform a detailed cost accounting as part of the next phase of the project.

There is also a risk that data might be biased by patients entering the system for the intervention alone and subsequently leaving the network afterwards. This is an important risk to consider especially with a major teaching hospital which draws many transient patients from out of state. However, our analysis of how long patients, both control and intervention, were in the healthcare network suggest this risk is minimal. Both sets of patients appear to be endogenous to the geographical area of the hospital and stable in the network, being within the system on average for greater than 4 years. The choice of the pre and post phase for the control group at the midpoint of their career in the health system was artificial and may have introduced a timing bias or sampled from periods of high or low utilization. However, those differences would be expected to average out across the population and over time. The rationale for this choice was to help capture both baseline behavior and potential secular changes over time. Because the average healthcare system career was over 4 years, patient entry dates were uniformly distributed across the entire sample period and since the pre-post interval was 2 years, it was felt this was a representative sample of the healthcare careers of both the intervention and control groups.

Patients who received the intervention were, in general, a high utilizing group. To control for regression to the mean and costs that might naturally decrease over time after patients had already been referred to specialists and received multiple tests, we matched this group to a similarly high utilizing control group and still noted significant decreases in utilization in the intervention group. Nonetheless, there may have been additional factors we could not account for. In addition, because we only followed patients out 1 year post-intervention we may not have seen the full effects of the intervention and whether or not the effects continued. There were indications in the data that this might be the case. This would bias against the effectiveness of the intervention.

Finally, there is the risk of statistical over-precision versus clinical meaning. With studies of large sample size, one can find statistically significant differences with no clinical meaning. For example, in the demographic sampling, done under rigorous conditions through the RPDR, we found some statistically significant differences in languages spoken, ethnic identification and marital status. These differences were typically less than 1% which we would suggest has no clinical significance.

### Policy Recommendation

The data suggests that the intervention should be applied to all at risk populations, since the intervention has minimal risk, minimal cost and yields substantial benefits for patients with a wide variety of illnesses. The long-term effect of these interventions on healthy populations is unclear, but the data suggests that mind body interventions should perhaps be instituted as a form of preventative care similar to vaccinations or driver education. Such interventions are likely to be useful in population management and supported self-care, have negligible risk and cost and may help reduce the demand curve in healthcare. While the risk benefit ratio of this intervention is very favorable to further elucidate the effect size a prospective evaluation is called for.
