# Correction: Relaxation Response and Resiliency Training and Its Effect on Healthcare Resource Utilization

**DOI:** 10.1371/journal.pone.0172874

**Published:** 2017-02-21

**Authors:** James E. Stahl, Michelle L. Dossett, A. Scott LaJoie, John W. Denninger, Darshan H. Mehta, Roberta Goldman, Gregory L. Fricchione, Herbert Benson

The Competing Interests statement is incorrect. The correct Competing Interests statement is: The following authors hold or have held positions at the Benson-Henry Institute for Mind Body Medicine at Massachusetts General Hospital, which is paid by patients and their insurers for running the SMART-3RP and related relaxation/mindfulness clinical programs, markets related products such as books, DVDs, CDs and the like, and holds a patent pending (PCT/US2012/049539 filed August 3, 2012) entitled "Quantitative Genomics of the Relaxation Response": JS, MD, JD, DM, RG, GF, HB.
